# Participating in the Illness Journey: Meanings of Being a Close Relative to an Older Person Recovering from Hip Fracture—A Phenomenological Hermeneutical Study

**DOI:** 10.3390/nursrep12040073

**Published:** 2022-10-13

**Authors:** Cecilia Segevall, Siv Söderberg

**Affiliations:** Department of Nursing Sciences, Mid Sweden University, 83140 Östersund, Sweden

**Keywords:** close relatives, hip fracture, interviews, lived experience, nursing, older people, phenomenological hermeneutics, recovery

## Abstract

When an older person suffers an acute event, such as a hip fracture, it influences the whole family. Research shows that while close relatives want to be a part of the older person’s life during recovery it is associated with a high perceived level of stress and burden. To provide in-depth knowledge of close relatives’ experiences in this situation, the aim of this study was to elucidate meanings of being a close relative to an older person recovering from hip fracture surgery. This study has a qualitative descriptive phenomenological hermeneutical design. Narrative interviews were conducted with ten close relatives. Analysis was conducted using phenomenological hermeneutical interpretation which provided a deeper understanding of the close relatives’ lived experiences of their older person’s recovery from hip fracture surgery. The structural analysis revealed two themes; “Participating in the illness journey”, which was constructed of the subthemes of facing the unimaginable yet expected, encountering healthcare personnel, and noticing recovery and “Putting oneself aside”, which was constructed of the subthemes of placing daily life on hold, giving support, and feeling concern and fear.

## 1. Introduction

When a person is affected by an acute event, such as a hip fracture, it influences the whole family. For older people, a hip fracture is described as an unexpected, life-altering event that disrupts their taken-for-granted daily life [1]. The event also impacts their close relatives, that is, people who are close to them emotionally [2]. Close relatives are highly valued during recovery after hip fracture surgery by both older people and healthcare personnel, as they provide help and support in the hospital and after discharge [1,3,4,5]. While close relatives want to be a part of the older person’s life and provide practical and emotional support during recovery [2], the time to prepare and develop skills that might be useful is short, due to the unexpected nature of the fracture and the short length of such hospital stays [6].

Every year, approximately 1.7 million hip fractures occur worldwide [7], and the mean age of people suffering a hip fracture is approximately 80 years of age [8]. The hip fracture affects the older person’s ability to perform activities of daily life, leaving them dependent on others—often their close relatives—during recovery [1]. Thus, a hip fracture is a common health issue that threatens older people’s health physically, psychologically, and socially [9].

Research on close relatives to older persons recovering from hip fracture is scant and primarily conducted within the quantitative research paradigm focusing on stress and perceived burden. It shows that being a close relative is a significant stressor for a long time after the older person’s hip fracture [10,11]. Stress is associated with grief from watching the older person’s health deteriorate [12], and concerns with how to help the older person mobilize and perform activities in daily life [10,13]. It is also associated with the immense impact helping or assisting with activities in daily life has on close relatives’ own life [14], involving how to find the time to keep the household running [10,13]. Economic aspects have also been shown to be a concern for close relatives during the older person’s recovery [11]. The increased levels of stress for close relatives can potentially have a negative effect on their health [14,15], especially for those who have to simultaneously deal with their own medical and personal issues [13].

Factors that influence perceptions of stress or burden are related to the age of the person suffering a hip fracture, as well as their pre-fracture functional status and post-operative complications. The age of the person helping or supporting also seems to be of importance [10], as well as the cognitive state of the person recovering from hip fracture surgery. Close relatives of cognitively alert older persons described having positive experiences during recovery and expressed pleasure in seeing them make progress compared to relatives of cognitively impaired older persons. By contrast, they expressed dissatisfaction with lack of support and felt solely responsible for protecting the older person’s interests regarding care and rehabilitation [16]. Additionally, supporting an older person during recovery after hip fracture surgery can be seen as the natural thing to do, resulting in them spending more time together and becoming closer [15]; however, when recovery is taking longer than expected, close relatives can start to lose faith and to experience caring for their loved one as a burden [2].

The body of knowledge on what it means to be a close relative to an older person recovering from hip fracture surgery is limited. However, the results of existing research are concerning in relation to the health of close relatives. This study provides knowledge of this phenomenon and has the potential to better meet the needs of close relatives.

The aim of this study was to elucidate meanings of being a close relative to an older person recovering from hip fracture surgery.

## 2. Materials and Methods

### 2.1. Design

This study has a lifeworld perspective, referring to the world we live in together with others. The perspective considers humans’ own experiences and understandings of themselves, their bodies, and the meaning their life situations hold for them. The lifeworld is taken for granted, i.e., it is the world we do not reflect upon [17]. A phenomenological hermeneutic method, inspired by the French philosopher Ricoeur (1976), was used to elucidate meanings of being a close relative to an older person recovering from hip fracture surgery. The method has been developed for nursing research by Lindseth and Norberg [18] and is useful for explicating people’s lived experiences of a phenomenon. The method builds on the beliefs of Ricoeur, who linked the lifeworld theory of phenomenology with a hermeneutical focus on interpretation and understanding [18]. This paper was designed and written according to the Consolidated Criteria for Reporting Qualitative Research (COREQ) guidelines [19].

### 2.2. Participants and Procedure

A purposive sample of 10 close relatives (7 women and 3 men) of previously healthy and independent older people recovering from hip fracture surgery participated in the study. The number of participants was guided by the concept of information power by Malterud et al. [20], which focuses on sample suitability and data quality to provide nuanced and rich descriptions. To be included in the study, the participants had to be a Swedish speaking close relative of an older person recovering from hip fracture surgery and who was willing to talk about their lived experience. The participants ranged between 35 and 83 years old (Md = 58). Four participants were retired and six worked. Three participants lived with the older person recovering from a hip fracture, five lived with their own families, and two lived alone. In this study, the older person recovering from hip fracture surgery decided who they considered to be a close relative; consequently, participants were spouses, adult children, and close friends.

The close relatives were recruited from participants in a previous study [21]. The older people in that study were asked if they had a close relative who would be interested in participating in a study about the experiences of being a close relative; these people left the name and phone number of a close relative. The older people informed their close relative of these actions, and by the time the first author contacted the participants to further inform them about the study and ask if they were willing to participate, they knew they would be contacted. Eleven close relatives were contacted by the first author, and 10 chose to participate. A time and place for the interview were scheduled primarily according to the participant’s wishes, but later also in accordance with the recommendations on social distancing due to the COVID-19 pandemic outbreak.

### 2.3. Interviews

Personal interviews with a narrative approach [22] were carried out with the participants. Narratives are stories collected from individuals about their lived experiences and may touch on the individual’s past, present, and future [22,23]. Data in this study were obtained using an interview guide on the lived experiences of being a close relative to an older person who was recovering from hip fracture surgery. The interview guide was based on prior research. Before the beginning of each interview, the first author repeated the information about the study to enable the participants to ask questions about their participation in the study. The interview guide consisted of three questions (see Table 1). Follow-up questions such as “Can you give an example?”, “Can you tell me more about that?”, and “How did that make you feel?” were asked when clarification was needed.

The interviews were conducted one on one by the first author between January and May 2020. The interviewer is a female PhD and registered nurse with extensive experience of orthopaedic nursing. The researcher had no prior relationship with the participants. The interviews took place approximately three weeks to two months after the older person had their hip fracture. Four interviews were conducted face to face in the participant’s home and in the first author’s office, and six interviews were conducted over the phone (a result of the COVID-19 pandemic). The interviews lasted between 20 and 55 min (Md = 36) and were audio-recorded and transcribed verbatim.

### 2.4. Analysis—The Phenomenological Hermeneutic Interpretation

The transcribed interviews were analysed with a phenomenological hermeneutic interpretation. This research method consists of three interrelated phases: naïve understanding, structural analysis, and comprehensive understanding. The analysis involves a dialectical movement between the whole of the text and its parts, as well as between understanding and explanation [18]. Furthermore, the analysis involves following the movement of the text, from what it says to what it talks about [24].

In the first phase, naïve understanding, we read the interview texts several times to grasp the immediate meaning of what it means to be a close relative to an older person recovering from hip fracture surgery. In this phase, the text was seen as a whole, which we then used to guide and later validate the structural analysis. In the second phase, structural analysis, we divided the texts into meaning units. We carefully condensed the meaning units to avoid losing relevant content, expressed them as clearly as possible using everyday language, and reflected on them in terms of their similarities and differences. The condensed meaning units were then grouped based on similarities into subthemes and themes to elucidate the phenomenon of being a close relative to an older person recovering from hip fracture surgery, as revealed by the text. In the final phase of the analysis, comprehensive understanding, we reflected on the results of the naïve understanding and the structural analysis in relation to the aim and the context of the study. We used relevant literature and our preunderstandings as nurse researchers to reach a comprehensive understanding of the participants’ lived experience of the phenomenon of interest [18]. To enhance rigour, both authors were involved in the analysis and different interpretations were discussed until we reached an understanding.

### 2.5. Ethical Considerations

This study was conducted in accordance with the declaration of Helsinki (1964). This declaration promotes respect for all human beings and protects their health and rights while participating in research [25]. Participants received oral and written information about the study before they gave their voluntary informed consent to participate in writing. The provided information included the aim of the study, what it means to participate, and the participant’s right to withdraw from the study at any time without giving a reason. The participants were guaranteed confidentiality and were assured that the findings would be presented without any identifying information. The study was approved by the Swedish Ethical Review Authority (Dnr 2016-154-31, Dnr 2019-02090).

## 3. Results

### 3.1. Naïve Understanding

It seems that being a close relative of an older person recovering from a hip fracture meant a rapid change in daily life due to the sudden nature of a hip fracture, a reminder that events can happen that result in putting plans on hold, learning new things, and taking on more responsibility. It seems that close relatives became protective of the older person and stayed involved to make sure things would work out as best they could. During the older person’s hospital stay, close relatives wanted to be in contact with the older person and with the healthcare personnel to receive information and discuss discharge plans. Close relatives also took actions to refurnish the home for easier living when the older person was discharged from hospital, something that was not always considered necessary by the older people. Being a close relative meant respecting and supporting the older person’s wishes to not have municipal social services, although they were aware that this decision meant an extra workload for them. It seems that close relatives considered helping the older person during a health crisis as an expected, obvious thing to do and to give something back. It was mentally tiring to try to be in two places at once and to feel that they were not doing enough. Support from others, finding time to continue doing things they enjoyed, and seeing the older person’s need for help during recovery as temporary seemed important.

### 3.2. Structural Analysis

The analysis revealed two themes, with six subthemes (see Table 2). It became evident that close relatives participated in the illness journey after the older persons hip fracture as they faced the unimaginable yet expected, encountering healthcare personnel as well as noticing the older person recover. While participating in the illness journey, close relatives put themselves aside, meaning they had to put their own lives on hold to give support to the older person, and at the same time feeling fear and concern for the future. In this section, the themes and subthemes for the close relatives are presented and illustrated with quotations from the interview texts.

#### 3.2.1. Participating in the Illness Journey

The theme ‘participating in the illness journey’ includes the following subthemes: facing the unimaginable yet expected; encountering healthcare personnel; and noticing recovery.

#### 3.2.2. Facing the Unimaginable yet Expected

The subtheme facing the unimaginable yet expected was related to close relatives’ immediate reactions when they heard about the fall. Close relatives were concerned about the extent of the injury, hoping that it was not too severe while realizing that it could be something serious. They felt overwhelmed, sad, worried, and responsible.

“*A girl came to me and said “you have to hurry, your dad has fallen.” And it was just no, no, no, this wasn’t supposed to happen and I hoped it was nothing. I mean I hoped it wasn’t something serious, but as I got out there and saw the leg pointing in a different direction it was just…Disaster*.”

“*I keep thinking it’s my fault. If I hadn’t driven him home to get the dessert he wouldn’t have slipped on the ice. He didn’t want to keep me waiting so he was in such a hurry to get back in the car so that we could drive back to my place.*”

Facing the unimaginable also involved feelings of relief, since the older person only suffered a hip fracture instead of being affected by a more severe illness that would forever change them, such as a stroke. The fall brought an awareness that life-changing events can happen, and it was a wake-up call to not take life for granted. This included an awareness that with the older person’s age, they would face the responsibility of supporting the older person at some point in the future. Up until now, they felt fortunate about the health and independence of the older person prior to the hip fracture while simultaneously realizing that the older person was not as strong as they wanted to appear

“*I think that as you get older, you realize that things will change for one reason or another. It’s a natural part of aging.*”

#### 3.2.3. Encountering Healthcare Personnel

The subtheme encountering healthcare personnel was related to meeting and experiencing healthcare personnel, whether themselves or through the older person. For close relatives, encountering healthcare personnel involved feelings of both satisfaction and disappointment. Feeling satisfied with healthcare personnel occurred when they were met in a friendly manner when they visited at the hospital or called, as well as when they received sufficient information that met their needs. In addition, close relatives’ satisfaction was related to the older person’s satisfaction with care, as well as being content with receiving information about care and plans solely from the older person.

“*I didn’t feel the need to have contact with healthcare staff, I got all the information I needed from him. He was content and felt well taken care of and that was enough for me.*”

In contrast, not being informed according to one’s needs led to close relatives feeling disappointed. To close relatives, lacking information meant lacking knowledge about the fracture, treatment, and prognosis. Despite their explicit desire to get in contact with the doctors, it seemed that contacting close relatives appeared to be unnecessary, leading to them feeling uniformed and uninvolved. Not wanting to be perceived as difficult meant that close relatives avoided being assertive and calling repeatedly until they received answers. Feeling uniformed and uninvolved meant wishing for a better dialogue with healthcare personnel in which the concerns they voiced were considered, especially regarding discharge planning. It also meant being surprised that discussions and decisions about the older person’s needs after discharge from the hospital was a matter limited exclusively for the older person and the healthcare personnel. Being left out of this discussion led to feelings of frustration with being unable to influence the decision despite being highly involved once the older person returned home.

“*I couldn’t believe it. He had told the discharge planning nurse that his daughters would help him at home and that he didn’t need municipal home services. No one asked for our opinion or explained what that involved.*”

For close relatives, feeling disappointed also involved a lack of complete trust in healthcare personnel. The hospital ward was perceived as disorganized as activities were not conducted as planned; for example, activities that were asked for, such as pain medication for the older person, were not attended to. In addition, close relatives’ disappointment was related to the older person’s negative experiences of the hospital stay and not feeling cared for.

“*She told me that she felt like a burden when she asked for something. I hope that I won’t have to feel that way if I need care.*”

#### 3.2.4. Noticing Recovery

The subtheme noticing recovery was related to seeing that the older person was becoming more independent and involved feeling hope for the future. It involved close relatives’ feelings of amazement due to the speedy recovery that enabled the older person to mobilize on their own, take longer walks, and participate in household chores—i.e., regaining independence and returning to life as prior to the hip fracture.

“*He keeps taking longer and longer walks every day. It’s good to see him doing better.*”

The older person becoming more independent meant less responsibility for close relatives, since assistance was not needed to the same extent as immediately after the homecoming. Having less responsibility was a relief and strengthened close relatives’ hope for the future. Feeling hope was partly based on close relatives’ previous experiences of people they knew who had suffered a hip fracture and had recovered, and also partly based on the belief that good physical condition was important for recovery. Close relatives felt grateful for the fact that the older person was in “good shape” prior to the hip fracture.

“*You see all the time that people get back after their hip fracture, so why shouldn’t he?*”

Feeling hope also meant an awareness of close relatives’ need to be patient, since healing and recovery take time. Feeling hope for the future was shadowed by uncertainties regarding the extent to which the older person’s age would affect the potential for a total recovery.

“*I mean, I don’t know…it’s the age thing. [Silence] I hope she recovers but I know there aren’t any guarantees.*”

While hope for a total recovery was desired by close relatives, feeling satisfied with less was also present if the older person was satisfied.

### 3.3. Putting Oneself Aside

The theme ‘putting oneself aside’ includes the following subthemes: placing daily life on hold; giving support; and feeling concern and fear.

#### 3.3.1. Placing Daily Life on Hold

The subtheme placing daily life on hold was related to close relatives putting their own lives and needs on pause for the older person’s benefit, on behalf of their own well-being. Being there for the older person entailed a great change in everyday life for close relatives and inevitably imposed a heavier workload. Close relatives attempted to manage their own lives while also being there for the older person, and evoked mixed feelings of managing and struggling. Managing meant being a good planner and being efficient and capable of dealing with the extra work that ‘being there’ required; struggling meant efforts to balancing time by taking time off from work or temporarily reducing working hours to manage. Some close relatives had to manage responsibility for not only one but two older persons; this was the case when the older person recovering from a hip fracture was the household’s primary caregiver.

“*My mother suffers from dementia, and my dad looks after her and keeps the household running. But when he got his hip fracture, he also needed to be taken care of. [Silence] That complicated things, because I wanted to be there for Dad when he was in hospital but at the same time I needed to look after Mom.*”

Putting daily life on hold also meant that close relatives had to ask for help themselves to deal with both the practical and emotional aspects of their own everyday lives. In addition, having someone to talk to when feeling the need to ‘spill their guts’ was invaluable to close relatives. Putting oneself aside involved feelings of reward for close relatives, but it was also undeniably demanding. Trying to balance one’s own life with being there for the older person was draining and tiring, and it impacted close relatives’ health as they experienced feeling ill and had difficulties with relaxing.

“*Perhaps it’s not such a good idea, trying to be in two places at once, I’m starting to feel really worn down.*”

Furthermore, close relatives felt grateful toward the people who were there for them in this difficult situation. Two factors that provided energy to continue helping during the older person’s recovery included believing that the hip fracture was temporary in nature and the older person deciding to accept help from the municipal home services.

#### 3.3.2. Giving Support 

The subtheme giving support was related to being there for the older person and helping with whatever, whenever; it involved an acknowledgement that they will do it again should it be needed. For close relatives, giving support was the obvious and natural thing to do and appeared to be intertwined with a sense of supporting out of duty and obligation; these functioned as their driving forces to give support. Seeing giving support as the obvious thing to do indicated a relationship of mutual give and take for close relatives and that, had the situation been reversed, the older person would not hesitate to do the same. Supporting out of obligation meant that giving support was the expected thing to do; for close relatives, this expectation meant that their support was expected to be needed at some point, given the older person’s age. In contrast, not being able to give support the way they wanted led to feeling guilty and was connected to close relatives’ own old age.

“*I mean, I’m over 80 so I can’t be there for him. I’m glad I manage myself. I feel sorry, but I can’t be a Florence Nightingale to him.*”

For close relatives, giving support meant preparing for the older person’s homecoming and adjusting the older person’s home to make it ‘easily accessible’. Preparing meant making alterations: removing rugs, taking out thresholds, and moving the bed to make it easy access in case the older person wanted to lay down and rest during the day. These alterations were not always made in consultation with the older person, nor were they initially wanted by the older person.

“*We discussed moving the bed from upstairs, but she didn’t want that and so I told her, “you need to realize that you won’t be able to walk up and down the stairs every time you want to lay down and rest,” and I guess she understood that, and so we moved the bed.*”

Giving support meant that close relatives took on the responsibility for a well-functioning everyday life, such as helping with mobilization and dressing, along with performing household chores. Taking responsibility for the household involved performing activities that under normal circumstances would be performed by the older person. Performing a different set of chores opened close relatives to being exposed to critique from the older person, who sometimes commented on the close relative’s performance. Being exposed to critique meant feeling inadequate and not good enough.

“*I believe that has to do with being a woman or a man. Women want the house to be clean and tidy. I feel satisfied with the result when I have cleaned. But my cleaning doesn’t live up to her expectations.*”

Giving support also meant being encouraging and letting the older person know they were making progress. Close relatives noticed that the older person’s mood had taken a turn for the worse, given the forced dependence that was brought on by the hip fracture. Witnessing the older person feeling “low” or “grumpy” was difficult for close relatives, and they encouraged the older person to try to live life as they did before the fracture even though it appeared to be difficult. It involved activities they enjoyed, and the mended hip fracture allowed, such as taking short walks and going on road trips.

“*I think it’s important that you try to get out and don’t get stuck sitting indoors. It might take a bit more effort, but in the end it’s worth it.*”

Giving support was also related to close relatives’ unawareness of what it actually meant to be a helper, and with time, it became difficult to continue supporting the older person to the extent that was needed. When trying to arrange formal help, they became aware that it was not as easy as they had expected, and they ultimately found themselves questioning whether their initial decision to help was the right one.

“*If I had known that it [organizing formal help] would be this difficult, I probably would have thought differently. For all our sakes, it probably would have been better if all this was organized at the hospital.*”

#### 3.3.3. Feeling Concern and Fear

The subtheme feeling concern and fear was related to worrying about the condition of the older person upon discharge and involved thoughts on how they would manage, as well as fearing another fall. For close relatives, feeling concern was related to feelings that the older person’s hospital stay was too short and involved a wish that the older person could have stayed another couple of days “just in case”.

“*I thought, no it’s too soon to be discharged already. I would have preferred it if she stayed another couple of days in case something unforeseen would happen*”

The “early” discharge brought mixed emotions of both happiness and concern for close relatives. Happiness stemmed from the feeling of not being alone in the house anymore and seemed to be an indicator that the older person was “well enough” to be discharged. Feeling concern was related to the older person’s condition when close relatives had not seen for themselves how the older person was doing, since they were not allowed at the hospital. For close relatives, not being allowed to visit was related to the restrictions on hospital visitors due to the COVID-19 pandemic, which made it difficult to stay connected to the older person during their hospital stay. Staying connected over the phone was not enough and meant that close relatives did not feel they were part of what happened at the hospital, could not see how the older person was managing, and could not ease their concerns regarding the older person’s well-being, mobility, and pain. Upon the older person’s homecoming, being able to see the older person meant feeling relived over the fact that she/he was in better condition and less upset than expected.

*“It felt good when she was discharged… you know, you get to see with your own eyes and form an opinion for yourself. It’s difficult to imagine over the phone.”*

For close relatives, feeling worry and fear meant a desire to stay close in case the older person needed assistance with something at home, day or night. During the night, this meant sleeping nearby and “light” to hear the older person. In the beginning, close relatives needed to get up and help the older person get to the bathroom during night, but as time passed, it was enough for close relatives to stay in bed and just listen that everything was okay. Sleeping nearby and “light” meant being sleep-deprived and involved feeling exhausted and not functioning well during the day as a result. Feeling worry and fear meant wanting to protect the older person to prevent additional injury. For close relatives, protecting meant reminding the older person to be careful and go slow during mobilization and reminding them to use their walking aids. Protecting also meant not wanting the older person to walk outdoors by themselves on icy ground and advising them to keep the municipal home services a while longer, just in case.

“*I’m so worried that he will fall again because it seems that he has forgotten that he recently suffered a hip fracture. He is in such a hurry to do things and I have to remind him to take it slow.*”

## 4. Comprehensive Understanding and Reflections

In the last phase of the interpretation process, comprehensive understanding, a reflective reading of the text as a whole is presented. The preunderstandings of the authors, the naïve understanding, and the results of the structural analysis are all taken into account so as to gain a deeper understanding [18]. This study suggests that meanings of being a close relative to an older person involve participating in the illness journey while putting oneself aside through the process of recovering from hip fracture surgery.

Participating in the illness journey while putting oneself aside during the older person’s recovery can be understood as a dramatic transition in daily life for close relatives and means facing the unimaginable yet expected event of a health crisis, feeling satisfied or disappointed with healthcare personnel, and (hopefully) being able to notice recovery. Furthermore, it means placing daily life on hold to support the older person while feeling concern and fear about the older person’s condition, as well as another fall. A transition can be described as a period between two relatively stable periods in life and involves adapting and adjusting to a new situation, such as participating in the illness journey while putting oneself aside. Transitions are characterized by uncertainty, change, and the disruption of daily life. People in transition tend to be vulnerable, which in turn may affect their health [26]. Participating in the illness journey while putting oneself aside during an older person’s recovery from hip fracture surgery can also be seen as an expression of an ethical demand. Ethical demands are unspoken in nature and are based on trust, love, and sympathy. Encountering another human being means to hold a part of the other’s life in our hands [27].

For close relatives in this study, facing the unimaginable yet expected means that they were aware that old age entails a growing risk for falling ill or being affected by injury, which could lead to needing help. We interpreted this to be an expression of the view that growing old is a natural part of aging that is associated with uncertainties regarding health and life. Growing old involves many life changes, including losses, limitations, and uncertainty. These make life more insecure, and dependence on others can become a reality, meaning that life cannot be taken for granted [28]. We saw the older person’s hip fracture as the starting point for the transitions of close relatives. Although a health crisis was to be expected at some point due to aging, close relatives were still emotionally unprepared for a hip fracture [26].

Encountering healthcare personnel is associated with contradictory feelings of satisfaction and disappointment and was connected to how close relatives felt they were treated by healthcare personnel. This can be seen as an expression that healthcare personnel have the power to confirm or disconfirm close relatives during their encounters. Close relatives felt satisfied, i.e., confirmed, when they were met in a friendly manner and received information that met their needs. In contrast, they felt disappointed, i.e., disconfirmed, when they were not informed according to their needs or not heard when they raised concerns. Confirmation entails being seen as a human being [29]. For close relatives, it is important to be confirmed, as this implies that they are part of a team with the ill person [30]. Confirmation is about viewing a family member as important, and when nurses confirm the family member, they listen and offer them comfort [31].

Close relatives in this study explicitly expressed wanting more information, but they were not accommodated. In situations where close relatives do not receive enough information about what is happening or about what the future holds, they can feel left out of the picture and neglected by healthcare personnel [32]. For the close relatives in this study, not having their expectations met around receiving information has the potential to harm their trust in healthcare personnel. According to Løgstrup [27], trust is fundamental to human existence: we believe and trust each other, and when one’s expectations are not met, trust is abused, which affects our future encounters. During a transition involving health and illness experiences, people need to learn and master new skills and behaviours, and nurses are usually the ones who facilitate the process of learning new things [26]. In situations where healthcare personnel do not involve close relatives, the completion of the transition may be delayed. Feeling disappointed also arises from not being involved in discussions about discharge planning for the older person’s needs after the hospital, even though they played a significant role in the older people’s lives when discharged. Not being involved can affect close relatives’ health due to increased stress [10] and limit their ability to support the older person’s recovery [30].

Seeing the older person recover—i.e., become more independent—provides feelings of hope for the future. Hope arises in conjunction with the future and change and can be seen as a freeing from something in the present; thus, hope provides a platform for taking the leap toward the future [33]. Hope has also been described as embedded in oneself, that remains positive no matter what happens [34]. Seeing the progress of recovery can also be a driving force to keep up one’s strength to continue helping [35]. Although close relatives hoped for a total recovery for the older person, they would be content if the older person was satisfied. The concept of recovery does not imply living a life without symptoms or limitations, but finding new ways to learn to live in a new way after an acute, life-altering event [36].

Placing daily life on hold means that close relatives disconnected with their ordinary course of life on behalf of the older person and the inevitable heavier workload. According to Løgstrup [27], every relationship with another person holds an unspoken demand, such as an ethical demand in encountering a person who needs one’s help. Putting oneself aside was interpreted as an expression of unselfishness that limited their freedom. Being unselfish can mean regarding one’s own needs as unimportant and focusing all attention on the needs of the ailing older person, and it involves losing one’s freedom to do things on one’s own [35]. Taking on a heavier workload affected close relatives’ well-being, as they were drained of energy and realized that it was not healthy to keep trying to be in two places at once. We interpreted this as close relatives being aware of the negative impact that caring/helping has on their own health but unable to do something about it. Ignoring one’s own needs and emotions can lead to feelings of also being affected by the illness and can impact close relatives’ health for the worse [32].

Giving support means that helping was the obvious and natural thing do to when the older person experienced a health crisis. For close relatives, taking on the responsibility to help the older person can be seen as the expression of an ethical demand. The ethical demand necessitates taking care of the other person’s life in the way that is best for them. Given the unspoken nature of the demand, and that the demands of human beings seldom coincide with others, there is an obvious risk that the two sets of demands will collide [27]; this seems to be the case for close relatives in this study. While helping the older person can be seen as a rather unselfish act that is performed with the best intentions, close relatives were exposed to critique, which led to feelings of not being good enough. We understood this as close relatives not being confirmed by the older person. Being confirmed is described as being acknowledged by an important other, and it evokes feelings of hope, comfort, and confidence [37]. According to Buber [29], everyone wishes to be confirmed, and not being confirmed can lead to loss of confidence and a distrust in one’s ability to help. Feeling confident is essential to maintaining capacity to provide care [38]. For the close relatives in this study, being critiqued while giving support led to feelings of not being good enough, as their actions did not live up to the older person’s expectations.

Although pleased about the discharge of the older person, close relatives also felt concern about how things would work out at home. They would have preferred the hospital stay to be longer for the older person to improve more. We interpreted this to be about close relatives’ own worry of not feeling ready and confident in their capacity for care. When close relatives experience a relative’s care transition, they worry about their capacity to manage, and they need time to prepare emotionally [39]. Given that hospital stays are shortening, close relatives now have less time to prepare for what is to come. When they do not feel prepared and they take on the responsibility to help (i.e., answer the ethical demand), they may be left with feelings of guilt that do not leave them [27]. Close relatives feared another fall and thus remained nearby ‘just in case’ and prompted the older person to take a slow and steady approach to mobilization. We understood the need to stay nearby as wanting to protect the older person from further injuries. Protecting an older person involves promoting, demanding, and facilitating courses of actions that benefit the older person’s health [39].

### Methodological Considerations

Although it provides some very useful data on this sparsely researched area, this study has limitations that must be considered in interpretations of the results. The number of participants was relatively small; however, they spoke willingly about their lived experiences, which contributed to richness and depth in the analysis. The sample size was guided by the concept of information power [20]. According to this, sample suitability and data quality are more important than the number of participants. The use of telephone interviews may have its limitations, due to the inability to establish face to face rapport between the researcher and the participant, as well as the inability to see and follow up on the participants’ facial expressions during the interview. However, participants were so forthcoming with their experiences that it is possible that the anonymity provided by the telephone provided a sense of comfort. One phone interview was conducted as a result of a participant’s choice, the others were a result of the recommendations on social distancing due to the COVID-19 outbreak.

This study uses a phenomenological hermeneutical approach to interpret the lived experiences of being a close relative to an older person recovering from hip fracture surgery. According to Ricoeur [24], a text can be interpreted in several ways, but not all interpretations are equally probable. The interpretation presented in this study is the one that we found to be the most probable. Additionally, the transcribed text must be interpreted for the meaning of lived experience to be elucidated [18]. The meaning of a phenomenon cannot exist without preunderstanding—i.e., the researchers’ preunderstanding is essential and therefore cannot be put aside or bracketed [18]. In this study, we were aware of our preunderstandings, and the interpretation was performed from the perspective of our experiences and understanding of close relatives’ experiences of their loved ones’ recovery. The findings from this study cannot be generalized, as this is not the purpose of this qualitative research; nevertheless, they can be transferred to similar situations if they are decontextualized from the current context [40].

## 5. Conclusions

This study shows that being a close relative to an older person recovering from hip fracture surgery means participating in the illness journey and being there for the older person (i.e., answering the ethical demand) while unselfishly putting oneself aside. Close relatives take on the responsibility to help the older person despite not being informed or included by healthcare personnel. It seems important, both for the older person’s recovery from hip fracture surgery, as well as for the health of close relatives, that the latter knows what to expect during recovery; not knowing limits close relatives’ ability to support the older person. Being there for the older person is the natural thing to do based on love, trust, and sympathy for the older person. However, it is demanding and emotionally tiring, it exposes them to feeling not good enough, and it can potentially have a negative impact on their health, which needs to be considered in discharge planning for an older person recovering from hip fracture surgery.

This study can be used in nursing education and in various healthcare settings, as it highlights the experiences of being a close relative—including their needs and resources—as they are important to the older person’s recovery. It seems pivotal that orthopaedic nurses have knowledge about what close relatives can experience in relation to the older person’s hip fracture, especially since this knowledge can facilitate nursing care while planning for discharge, which should also consider the close relative’s role in the recovery process. It is also important to recognize that close relatives vary in their capacities and preferences for being involved in the older person’s recovery.

## Figures and Tables

**Table 1:** Interview guide.

1. Please tell about your relationship with the older person recovering from hip fracture surgery. 2. Please tell about how you experienced the older person’s hip fracture. 3. Please tell about how your life is today related to the older persons recovery.

**Table 2:** Overview of the themes and subthemes of the interview Texts of Close Relatives (*n* = 10).

Theme	Subtheme
Participating in the illness journey	Facing the unimaginable yet expected
	Encountering healthcare personnel
	Noticing recovery
Putting oneself a side	Placing daily life on hold
	Giving support
	Feeling concern and fear

## Data Availability

The data presented in this study are available on request from the corresponding author.
